# A Partial Repertory of Hypochondriasis

**Published:** 1888-11

**Authors:** N. W. Vandenburg

**Affiliations:** Fort Edward, N. Y.


### A PARTIAL REPERTORY OF HYPOCHONDRIASIS.

N. W. VANDENBURG, A. M., M. D., FORT EDWARD, N. Y.

(*a*.) *Hypochondrial anxiety.*

Æthusa, great sadness when alone; hallucinations.

Agn-c., despairing anxiety and sadness; peevish.

Aloe-soc., hypochron. worse in cloudy weather; hates and repels every one.

Alumina, trifles seem immense; dread of death with thoughts of suicide.

Ambra, great sadness and despair; fears becoming crazy.

Am-m., gloomy, apprehensive; hates involuntarily certain persons.

*Anac., hypoch. in forenoon; despairs of ever being able to do anything.

Arg-n., believes he is despised; that he will fail in his work.

Arn., hypoch. anxiety; peevish, morose, taciturn.

Ars., fears to be left alone lest he should harm himself; very restless.

Asafœt., hysterical anxiety, sad, fickle.

*Aur-m., full of fears, weary of life; desire of suicide.

Baryt-c., sadness, grief over trifles (Ac.); pusillanimous.

Bry., depressed, morose (Nux-v.), wishes to be alone; better in open air.

Cact., believes his malady is incurable (Ars.); consolation aggravates.

*Calc-c., feels as if some misfortune was about to happen (Cup.).

Caust., hopeless, full of timorous fancies; taciturn (Bry., Nux-v.).

Cham., very uncivil and impatient; everything goes too slowly.

Chel., feels as if she had committed a crime.

China, fixed idea that he is unhappy; discouraged.

Con., indifference; wants no one, yet does not want to be alone.

Cup-m., paroxysms of anxiety; fears he will lose his reason.

Graph., changeable, slow of thought; forgetful, forlorn.

Hell., indifferent, despairing, homesick; hates consolation (Cact.).

Hepar, low spirited, even to thoughts of suicide; weeping.

Ignat., from grief at loss of friends; taciturn.

Iod., low spirits; fear of evil with over-carefulness.

Kali-c., despondent in the open air (Bry.); fear of being alone (Ars.).

Lach., sadness, worse after sleep; suicidal.

Lil-tig., tormented about her salvation; fears insanity (Ambra, Cup.).

Lyc., satiety of life, particularly mornings in bed.

Nat-c., aversion to man and society; malicious.

Nat-m., enraged at consolation; desire to be alone.

*Nit-ac., vindictive; attacks of rage.

*Nux-v., cannot bear reading or conversation; wants to be alone.

Phos., anxious and restless at twilight; when alone.

*Phos-ac., depressed, weeping, homesick; night-sweats.

Phyt., melancholy, gloomy; indifference to life.

Plat., mania either with pride or changing to fear of men.

Podo., imagines he is to be very ill; gastric trouble.

Puls., silent, disgusted with everything; tearful moods.

Rhus-t., anxious, timid; worse at twilight (Phos.); tearful, restless.

*Sabad, melancholy from deep-seated abdominal irritation.

Sepia, indifferent to family and friends (Nez.); dreads to be alone; Kali-c., Ars.

Sil., desponding, melancholy, tired of life, easily crossed.

Staphis., from sexual excesses; sensitive to the least word.

Stann., feels like crying all the time, but it makes her worse.

Sulph., melancholy during the day; merry in the evening.

(*b*.) *Fears death.* Æthusa, of approaching death.

Alum., with thoughts of suicide.

Ars., when alone (Kal. c.); on going to bed (Lach.).

Cact., believes his disease incurable (Ars.).

Nit-ac., with anxiety about his disease (Ars.).

Phyt., great fear, sure he will die (Ac., Ars.).

Podo., imagines he is going to die.

Puls., tremulous anguish, as if death were near.

Rhus-t., satiety of life, with fear of death.

Sil., feels as if she would die.

Zinc, thinks of death calmly; or with fear, during pressure in the spine.

(*c*.) *Fears becoming crazy.*
Ambra, with diabolical faces crowding upon her fancy.
Calc-c., or that people will observe her confusion.
Chel., and forgets what she has done, or wants to do.
Cup-met., as if some misfortune were impending.
Iod., and the brain feels as if stirred up.
Lil-tig., crazy feeling on top the head.
(*d*.) *Thoughts of suicide.*
Alum., with dread of death.
Ant-c., by shooting.
Arg-n., thinks of killing himself.
Aur-met., desires suicide.
Cinch-off., desires to destroy himself, but lacks the courage.
Lach., tired of life, suicidal mood.
Nat-sul., must use self-control to prevent shooting himself.
Nux-v., thoughts of suicide, but is afraid to die.
Puls., anguish about the heart, even to suicide.
Rhus-t., wants to drown himself.
Sil., tired of life; wishes to drown herself.

*Wishes to be alone:* Aloe, Bry., Ign., Kali-c., Lyc., Nat-c., Nat-m., Nux-v., Rhus-t., Sul.

*Dislikes to be alone:* Æth., Ars., Caust., Con., Phos., Sepia.

Desires sympathy: Puls.

Hates sympathy: Cact., Hell., Nat-m.

Time passes too slowly: Alum., Arg-nit., Cham., Nux-v.

Times passes too quickly: Cocc., Therid.

Aversion to labor: Arn., Anac., Calc-c., Graph., Kali-c., Nux-v., Sep., Sulph.

Worse in the morning: Amm-m., Anac., Graph., Lach., Lyc., Nat-sul., Nux-v., Puls.

Worse during the day: Sulph., Zinc.

Worse during the evening and at night: Æth., Ars., Calc-c., Con., Hepar, Nat-c., Rhus, Puls.

